# Highly accurate protein structure prediction for the human proteome

**DOI:** 10.1038/s41586-021-03828-1

**Published:** 2021-07-22

**Authors:** Kathryn Tunyasuvunakool, Jonas Adler, Zachary Wu, Tim Green, Michal Zielinski, Augustin Žídek, Alex Bridgland, Andrew Cowie, Clemens Meyer, Agata Laydon, Sameer Velankar, Gerard J. Kleywegt, Alex Bateman, Richard Evans, Alexander Pritzel, Michael Figurnov, Olaf Ronneberger, Russ Bates, Simon A. A. Kohl, Anna Potapenko, Andrew J. Ballard, Bernardino Romera-Paredes, Stanislav Nikolov, Rishub Jain, Ellen Clancy, David Reiman, Stig Petersen, Andrew W. Senior, Koray Kavukcuoglu, Ewan Birney, Pushmeet Kohli, John Jumper, Demis Hassabis

**Affiliations:** 1 DeepMind, London, UK; 2 European Molecular Biology Laboratory, European Bioinformatics Institute, Hinxton, UK

**Keywords:** Proteomic analysis, Machine learning, Protein structure predictions, Structural biology

## Abstract

Protein structures can provide invaluable information, both for reasoning about biological processes and for enabling interventions such as structure-based drug development or targeted mutagenesis. After decades of effort, 17% of the total residues in human protein sequences are covered by an experimentally determined structure^1^. Here we markedly expand the structural coverage of the proteome by applying the state-of-the-art machine learning method, AlphaFold^2^, at a scale that covers almost the entire human proteome (98.5% of human proteins). The resulting dataset covers 58% of residues with a confident prediction, of which a subset (36% of all residues) have very high confidence. We introduce several metrics developed by building on the AlphaFold model and use them to interpret the dataset, identifying strong multi-domain predictions as well as regions that are likely to be disordered. Finally, we provide some case studies to illustrate how high-quality predictions could be used to generate biological hypotheses. We are making our predictions freely available to the community and anticipate that routine large-scale and high-accuracy structure prediction will become an important tool that will allow new questions to be addressed from a structural perspective.

## Abstract (web summary)

AlphaFold is used to predict the structures of almost all of the proteins in the human proteome—the availability of high-confidence predicted structures could enable new avenues of investigation from a structural perspective.

## Main

The monumental success of the human genome project revealed new worlds of protein-coding genes, and many researchers set out to map these proteins to their structures^3,4^. Thanks to the efforts of individual laboratories and dedicated structural genomics initiatives, more than 50,000 human protein structures have now been deposited, making *Homo sapiens* by far the best represented species in the Protein Data Bank (PDB)^5^. Despite this intensive study, only 35% of human proteins map to a PDB entry, and in many cases the structure covers only a fragment of the sequence^6^. Experimental structure determination requires overcoming many time-consuming hurdles: the protein must be produced in sufficient quantities and purified, appropriate sample preparation conditions chosen and high-quality datasets collected. A target may prove intractable at any stage, and depending on the chosen method, properties such as protein size, the presence of transmembrane regions, presence of disorder or susceptibility to conformational change can be a hindrance^7,8^. As such, full structural coverage of the proteome remains an outstanding challenge.

Protein structure prediction contributes to closing this gap by providing actionable structural hypotheses quickly and at scale. Previous large-scale structure prediction studies have addressed protein families^9–12^, specific functional classes^13,14^, domains identified within whole proteomes^15^ and, in some cases, full chains or complexes^16,17^. In particular, projects such as the SWISS-MODEL Repository, Genome3D and ModBase have made valuable contributions by providing access to large numbers of structures and encouraging their free use by the community^17–19^. Related protein bioinformatics fields have developed alongside structure prediction, including protein design^20,21^, function annotation^22–24^, disorder prediction^25^, and domain identification and classification^26–28^. Although some of our analyses are inspired by these previous studies, here we focus mainly on structural investigations for which scale and accuracy are particularly beneficial.

Structure prediction has seen substantial progress in recent years, as evidenced by the results of the biennial Critical Assessment of protein Structure Prediction (CASP)^29,30^. In particular, the latest version of AlphaFold was entered in CASP14 under the team name ‘AlphaFold2’. This system used a completely different model from our CASP13 entry^31^, and demonstrated a considerable improvement over previous methods in terms of providing routinely high accuracy^29,30^. Backbone predictions with sub-Ångström root mean square deviation (Cα r.m.s.d.) are now common, and side chains are increasingly accurate^2^. Good results can often be achieved even for challenging proteins without a template structure in the PDB, or with relatively few related sequences to build a multiple sequence alignment (MSA)^2^. These improvements are important, because more accurate models permit a wider range of applications: not only homology search and putative function assignment, but also molecular replacement and druggable pocket detection, for instance^32–34^. In light of this, we applied the current state-of-the-art method—AlphaFold—to the human proteome. All of our predictions can be accessed freely at https://alphafold.ebi.ac.uk/, hosted by the European Bioinformatics Institute.

## Model confidence and added coverage

We predicted structures for the UniProt human reference proteome (one representative sequence per gene), with an upper length limit of 2,700 residues^6^. The final dataset covers 98.5% of human proteins with a full chain prediction.

**Fig. 1:**
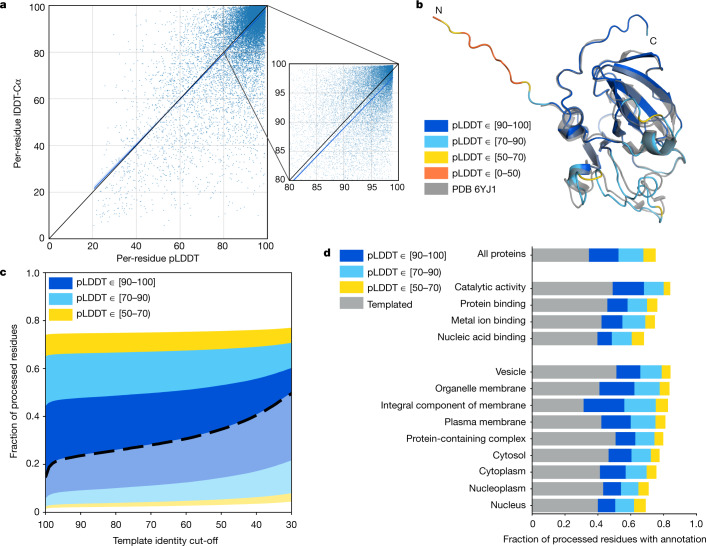
Model confidence and added coverage. **a**, Correlation between per-residue pLDDT and lDDT-Cα. Data are based on a held-out set of recent PDB chains (Methods) filtered to those with a reported resolution of <3.5 Å (*n* = 10,215 chains and 2,756,569 residues). The scatterplot shows a subsample (1% of residues), with the blue line showing a least-squares linear fit and the shaded region a 95% confidence interval estimated with 1,000 bootstrap samples. The black line shows *x* = *y*, for comparison. The smaller plot is a magnified region of the larger one. On the full dataset, the Pearson’s *r* = 0.73 and the least-squares linear fit is *y* = (0.967 ± 0.001) × *x* + (1.9 ± 0.1). **b**, AlphaFold prediction and experimental structure for a CASP14 target (PDB: 6YJ1)^64^. The prediction is coloured by model confidence band, and the N terminus is an expression tag included in CASP but unresolved in the PDB structure. **c**, AlphaFold model confidence on all residues for which a prediction was produced (*n* = 10,537,122 residues). Residues covered by a template at the specified identity level are shown in a lighter colour and a heavy dashed line separates these from residues without a template. **d**, Added residue-level coverage of the proteome for high-level GO terms, on top of residues covered by a template with sequence identity of more than 50%. Based on the same human proteome dataset as in **c** (*n* = 10,537,122 residues).

**Extended Data Fig. 1:**
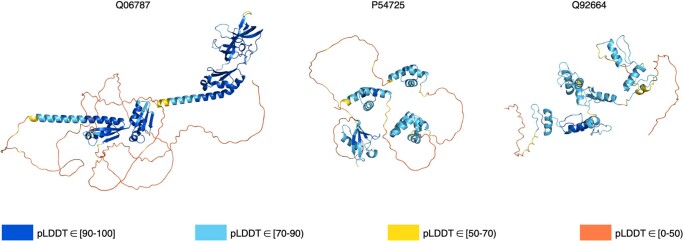
Example full chain outputs containing both high- and low-confidence regions. Q06787 (synaptic functional regulator FMR1) and P54725 (UV excision repair protein RAD23 homologue A) are predicted to be disordered outside the experimentally determined regions by MobiDB^73^. Q92664 (transcription factor IIIA) has been described as ‘beads on a string’, consisting of zinc-finger domains joined by flexible linkers^74^.

**Extended Data Fig. 2:**
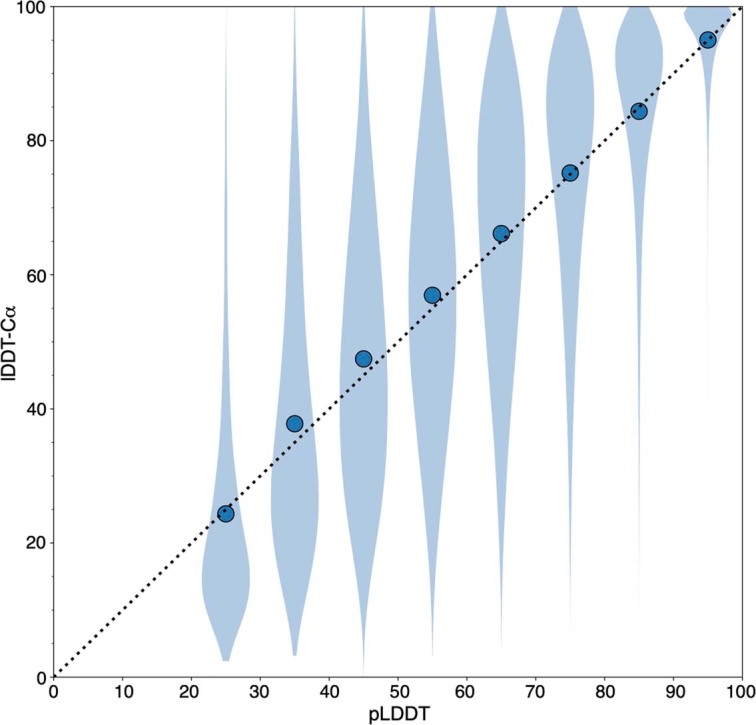
Distribution of per-residue lDDT-Cα within eight pLDDT bins. This represents an alternative visualization to Fig. 1a that does not sample the data. It uses the recent PDB dataset (Methods), which is restricted to structures with a reported resolution of <3.5 Å (*n* = 2,756,569 residues). Residues were assigned to bins of width 10 based on their pLDDT (minimum, 20; maximum, 100). Markers show the mean lDDT-Cα within each bin, while the lDDT-Cα distribution is visualized as a Matplotlib violin plot (kernel density estimate bandwidth, 0.2). The smallest sample size for the corresponding violin is 5,655 residues for the left-most bin.

For the resulting predictions to be practically useful, they must come with a well-calibrated and sequence-resolved confidence measure. The latter point is particularly important when predicting full chains, as we expect to see high confidence on domains but low confidence on linkers and unstructured regions (Extended Data Fig. 1). To this end, AlphaFold produces a per-residue confidence metric called predicted local distance difference test (pLDDT) on a scale from 0 to 100. pLDDT estimates how well the prediction would agree with an experimental structure based on the local distance difference test Cα (lDDT-Cα)^35^. It has been shown to be well-calibrated (Fig. 1a, Extended Data Fig. 2 and Extended Data Table 1) and full details on how the pLDDT is produced are given in the supplementary information of the companion AlphaFold paper^2^.

**Extended Data Fig. 3:**
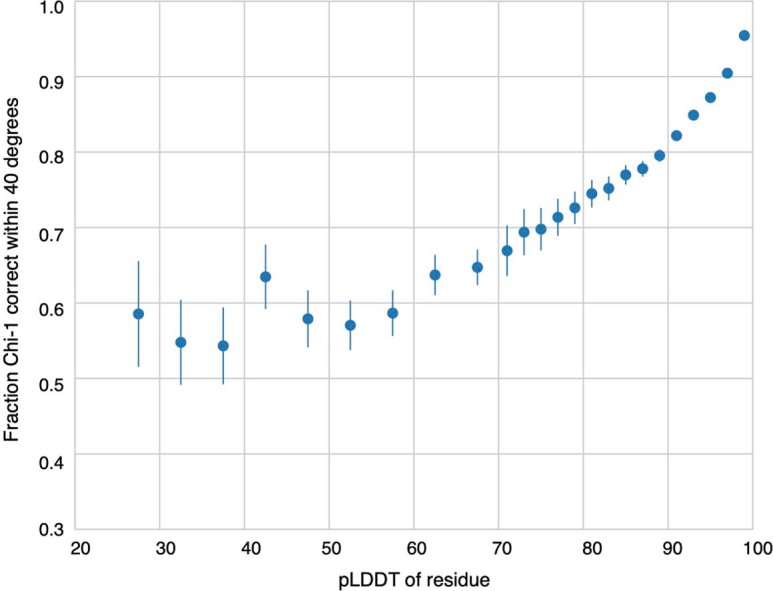
Relationship between pLDDT and side-chain *χ*_1_ correctness. Evaluated on the recent PDB dataset (Methods), which is restricted to structures with a reported resolution of <2.5 Å (*n* = 5,983 chains) and residues with a *B*-factor of <30 Å^2^ (*n* = 609,623 residues). Residues are binned by pLDDT, with bin width 5 between 20 and 70 pLDDT and bin width 2 above 70 pLDDT. A *χ*_1_ angle is considered correct if it is within 40° of its value in the PDB structure^75^. Markers show the proportion of correct *χ*_1_ angles within each bin; error bars indicate the 95% confidence interval (two-sided Student’s *t*-test). The smallest sample size for the error bars is 193 residues for the left-most bin.

We consider a prediction highly accurate when—in addition to a good backbone prediction—the side chains are frequently correctly oriented. On this basis, pLDDT > 90 is taken as the high accuracy cut-off, above which AlphaFold *χ*_1_ rotamers are 80% correct for a recent PDB test dataset (Extended Data Fig. 3). A lower cut-off of pLDDT > 70 corresponds to a generally correct backbone prediction (Extended Data Table 2). The accuracy of AlphaFold within a number of pLDDT bands is illustrated for an example protein in Fig. 1b.

Of the human proteome, 35.7% of total residues fall within the highest accuracy band (corresponding to 38.6% of residues for which a prediction was produced) (Fig. 1c). This is double the number of residues covered by an experimental structure. In total, 58.0% of residues were predicted confidently (pLDDT > 70), indicating that we also add substantial coverage for sequences without a good template in PDB (with a sequence identity below 30%). At the per-protein level, 43.8% of proteins have a confident prediction on at least three quarters of their sequence, while 1,290 proteins contain a substantial region (more than 200 residues) with pLDDT ≥ 70 and no good template.

The dataset adds high-quality structural models across a broad range of Gene Ontology (GO) terms^36,37^, including pharmaceutically relevant classes such as enzymes and membrane proteins^38^ (Fig. 1d). Membrane proteins, in particular, are generally underrepresented in the PDB because they have historically been challenging experimental targets. This shows that AlphaFold is able to produce confident predictions even for protein classes that are not abundant within its training set.

**Extended Data Fig. 4:**
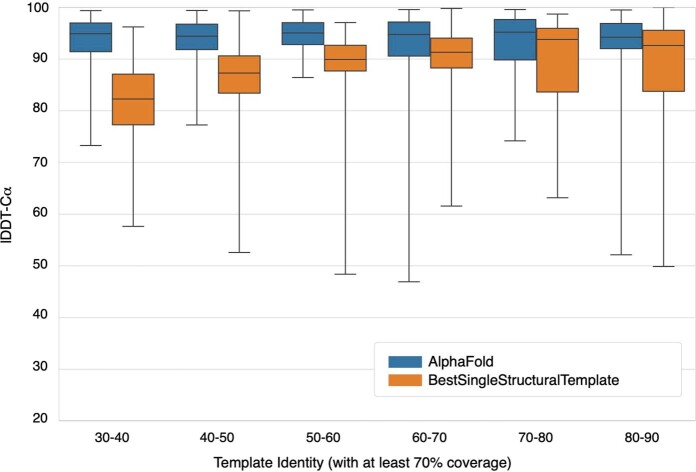
AlphaFold performance at a range of template sequence identities. lDDT-Cα for AlphaFold and BestSingleStructuralTemplate on 1 year of CAMEO targets^39^. Targets are binned according to the sequence identity of the best template covering at least 70% of the target, and a box plot is shown for each bin. The horizontal line indicates the median, boxes range from the lower to the upper quartile, and the whiskers extend from the minimum to the maximum. In total, 428 targets are included (see Source Data); the smallest number of targets in any bin is 18 Source data.

We note that the accuracy of AlphaFold was validated in CASP14^2^, which focuses on challenging proteins that are dissimilar to structures already available in the PDB. By contrast, many human proteins have templates with high sequence identity. To evaluate the applicability of AlphaFold to this collection, we predicted structures for 1 year of targets from the Continuous Automated Model Evaluation (CAMEO) benchmark^39,40^—a structure-prediction assessment that measures a wider range of difficulties. We find that AlphaFold adds substantial accuracy over the BestSingleStructuralTemplate baseline of CAMEO across a wide range of levels of template identity (Extended Data Fig. 4).

## Prediction of full-length protein chains

Many previous large-scale structure prediction efforts have focused on domains—regions of the sequence that fold independently^9–11,15^. Here we process full-length protein chains. There are several motivations for this. Restricting the prediction to pre-identified domains risks missing structured regions that have yet to be annotated. It also discards contextual information from the rest of the sequence, which might be useful in cases in which two or more domains interact substantially. Finally, the full chain approach lets the model attempt an inter-domain packing prediction.

**Fig. 2:**
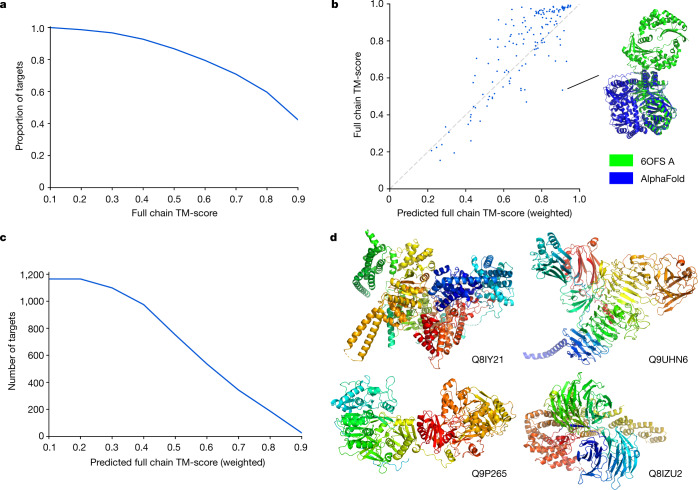
Full chain structure prediction. **a**, TM-score distribution for AlphaFold evaluated on a held-out set of template-filtered, long PDB chains (*n* = 151 chains). Includes recent PDB proteins with more than 800 resolved residues and best 50% coverage template below 30% identity. **b**, Correlation between full chain TM-score and pTM on the same set (*n* = 151 chains), Pearson’s *r* = 0.84. The ground truth and predicted structure are shown for the most over-optimistic outlier (PDB: 6OFS, chain A). **c**, pTM distribution on a subset of the human proteome that we expect to be enriched for structurally novel multidomain proteins (*n* = 1,165 chains). Human proteome predictions comprise more than 600 confident residues (more than 50% coverage) and no proteins with 50% coverage templates. **d**, Four of the top hits from the set shown in **c**, filtering by pTM > 0.8 and sorting by number of confident residues. Proteins are labelled by their UniProt accession. For clarity, regions with pLDDT < 50 are hidden, as are isolated smaller regions that were left after this cropping.

Inter-domain accuracy was assessed at CASP14, and AlphaFold outperformed other methods^41^. However, the assessment was based on a small target set. To further evaluate AlphaFold on long multi-domain proteins, we compiled a test dataset of recent PDB chains that were not in the training set of the model. Only chains with more than 800 resolved residues were included, and a template filter was applied (Methods). Performance on this set was evaluated using the template modelling score (TM-score^42^), which should better reflect global, as opposed to per-domain, accuracy. The results were encouraging, with 70% of predictions having a TM-score > 0.7 (Fig. 2a).

The supplementary information of the companion AlphaFold paper^2^ describes how a variety of useful predictors can be built on top of the main model. In particular, we can predict the residues that are likely to be experimentally resolved, and use them to produce a predicted TM-score (pTM), in which the contribution of each residue is weighted by the probability of it being resolved (Supplementary Methods 1). The motivation for the weighting is to downweight unstructured parts of the prediction, producing a metric that better reflects the confidence of the model about the packing of the structured domains that are present. On the same recent PDB test dataset, pTM correlates well with the actual TM-score (Pearson’s *r* = 0.84) (Fig. 2b). Notably, although some outliers in this plot are genuine failure cases, others appear to be plausible alternate conformations (for example, 6OFS chain A^43^ in Fig. 2b).

We computed pTM scores for the human proteome, in an effort to identify multi-domain predictions that could feature novel domain packings. The criteria applied were a pLDDT > 70 on at least 600 residues constituting over half the sequence, with no template hit covering more than half the sequence. The distribution of pTM scores after applying the above filters is shown in Fig. 2c. Note that we would not expect uniformly high TM-scores to be achievable for this set, as some proteins will contain domains that are mobile relative to each other, with no fixed packing. Of the set, 187 proteins have pTM > 0.8 and 343 have pTM > 0.7. Although we expect the inter-domain accuracy of AlphaFold to be lower than its within-domain accuracy, this set should nonetheless be enriched for interesting multi-domain predictions, suggesting that the dataset provides on the order of hundreds of these. Four examples—the predictions with the highest number of confident residues subject to pTM > 0.8—are shown in Fig. 2d.

## Highlighted predictions

We next discuss some case study predictions and the insights that they may provide. All predictions presented are de novo, lacking any template with 25% sequence identity or more covering 20% of the sequence. Our discussion concerns biological hypotheses, which would ultimately need to be confirmed by experimental studies.

### Glucose-6-phosphatase

**Fig. 3:**
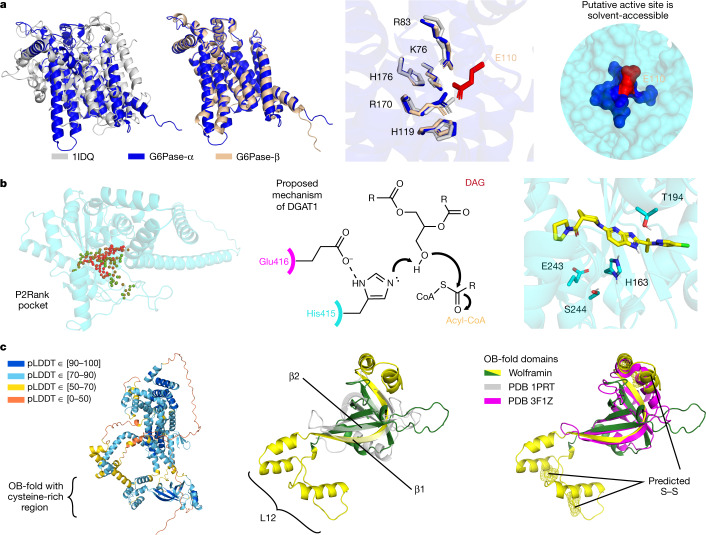
Highlighted structure predictions. **a**, Left, comparison of the active sites of two G6Pases (G6Pase-α and G6Pase-β) and a chloroperoxidase (PDB 1IDQ). The G6Pases are glucose-forming enzymes that contain a conserved, solvent-accessible glutamate (red; right) opposite the shared active-site residues (middle). **b**, Left, pocket prediction (P2Rank^65^) identifies a putative binding pocket for DGAT2, which is involved in body-fat synthesis. Red and green spheres represent the ligandability scores by P2Rank of 1 and 0, respectively. Middle, a proposed mechanism for DGAT1^51^ activates the substrate with Glu416 and His415, which have analogous residues in the DGAT2 pocket. The docked inhibitor is well placed for polar interactions with His163 and Thr194 (right). The chemical structure (middle) is adapted from ref. ^51^. **c**, Predicted structure of wolframin, mutations in which cause Wolfram syndrome. Although there are regions in wolframin with low pLDDT (left), we could identify an OB-fold region (green/yellow), with a comparable core to a prototypical OB-fold (grey; middle). However, the most similar PDB chain (magenta; right) lacks the conserved cysteine-rich region (yellow) of our prediction. This region forms the characteristic β1 strand and an extended L12 loop, and is predicted to contain three disulfide bridges (yellow mesh).

G6Pase-α (UniProt P35575) is a membrane-bound enzyme that catalyses the final step in glucose synthesis; it is therefore of critical importance to maintaining blood sugar levels. To our knowledge, no experimental structure exists, but previous studies have attempted to characterize the transmembrane topology^44^ and active site^45^. Our prediction has very high confidence (median pLDDT of 95.5) and gives a nine-helix topology with the putative active site accessible via an entry tunnel that is roughly in line with the surface of the endoplasmic reticulum (Fig. 3a and Supplementary Video 1). Positively charged residues in our prediction (median pLDDT of 96.6) align closely with the previously identified active site homologue in a fungal vanadium chloroperoxidase (PDB 1IDQ; r.m.s.d. of 0.56 Å; 49 out of 51 aligned atoms)^46^. As these enzymes have distinct functions, we investigated our prediction for clues about substrate specificity. In the G6Pase-α binding pocket face, opposite the residues shared with the chloroperoxidase, we predict a conserved glutamate (Glu110) that is also present in our G6Pase-β prediction (Glu105) but not in the chloroperoxidase (Fig. 3a). The glutamate could stabilize the binding pocket in a closed conformation, forming salt bridges with positively charged residues there. It is also the most solvent-exposed residue of the putative active site, suggesting a possible gating function. To our knowledge, this residue has not been discussed previously and illustrates the novel mechanistic hypotheses that can be obtained from high-quality structure predictions.

### Diacylglycerol *O*-acyltransferase 2

**Extended Data Fig. 5:**
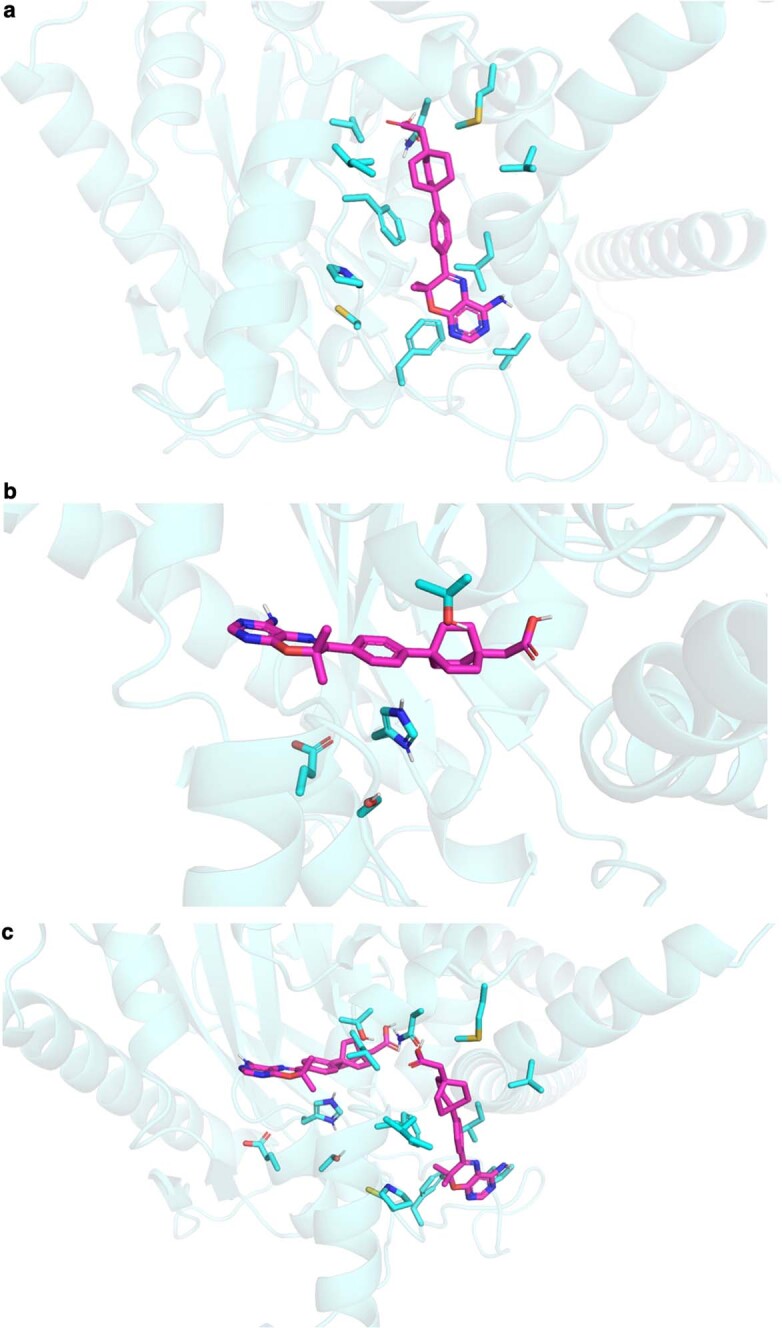
Docking poses for a DGAT1-specific inhibitor in DGAT2. **a**, Top binding pose from Autodock Vina for a DGAT1-specific inhibitor in DGAT2, which does not match the predicted binding pocket for a DGAT2-specific inhibitor. **b**, Next best binding pose, which matches the binding pocket for the DGAT2-specific inhibitor, but does not contain components that satisfy the polar side chains His163 and Thr194. **c**, Relative positions of both binding poses.

Triacylglycerol synthesis is responsible for storing excess metabolic energy as fat in adipose tissue. DGAT2 (UniProt Q96PD7) is one of two essential acyltransferases catalysing the final acyl addition in this pathway, and inhibiting DGAT2 has been shown to improve liver function in mouse models of liver disease^47^. With our highly confident predicted structure (median pLDDT of 95.9), we set out to identify the binding pocket for a known inhibitor, PF-06424439 (ref. ^48^). We identified a pocket (median pLDDT of 93.7) in which we were able to dock the inhibitor and observe specific interactions (Fig. 3b) that were not recapitulated in a negative example^49^ (Extended Data Fig. 5 and Supplementary Methods 2). DGAT2 has an evolutionarily divergent but biochemically similar analogue, diacylglycerol *O*-acyltransferase 1 (DGAT1)^50^. Within the binding pocket of DGAT2, we identified residues (Glu243 and His163) (Fig. 3b) that are analogous to the proposed catalytic residues in DGAT1 (His415 and Glu416)^51^, although we note that the nearby Ser244 in DGAT2 may present an alternative mechanism through an acyl-enzyme intermediate. Previous experimental research with DGAT2 has shown that mutating His163 has a stronger deleterious effect than mutating a histidine that is two residues away^52^. Additionally, Glu243 and His163 are conserved across species^50^, supporting this hypothesized catalytic geometry.

### Wolframin

Wolframin (UniProt O76024) is a transmembrane protein localized to the endoplasmic reticulum. Mutations in the *WFS1* gene are associated with Wolfram syndrome 1, a neurodegenerative disease characterized by early onset diabetes, gradual visual and hearing loss, and early death^53,54^. Given the lower confidence in our full prediction (median pLDDT of 81.7) (Fig. 3c), we proposed identifying regions that are unique to this structure. A recent evolutionary analysis suggested domains for wolframin, which our prediction largely supports^55^. An interesting distinction is the incorporation of a cysteine-rich domain (Fig. 3c, yellow) to the oligonucleotide binding (OB) fold (Fig. 3c, green and yellow) as the characteristic β1 strand^56^. The cysteine-rich region then forms an extended L12 loop with two predicted disulfide bridges, before looping back to the prototypical β2 strand. Comparing our prediction for this region (median pLDDT of 86.0) to existing PDB chains using TM-align^42,57^ identified 3F1Z^58^ as the most similar known chain (TM-score of 0.472) (Fig. 3c, magenta). Despite being the most similar chain, 3F1Z lacks the cysteines that are present in wolframin, which could form disulfide cross-links in the endoplasmic reticulum^59^. As this region is hypothesized to recruit other proteins^55^, these structural insights are probably important to understanding its partners.

## Regions without a confident prediction

As we are applying AlphaFold to the entire human proteome, we would expect a considerable percentage of residues to be contained in regions that are always or sometimes disordered in solution. Disorder is common in the proteomes of eukaryotes^60,61^, and one previous study^62^ estimated that the percentage of disordered residues in the human proteome is between 37% and 50%. Thus disorder will have a large role when we consider a comprehensive set of predictions that covers an entire proteome.

**Fig. 4:**
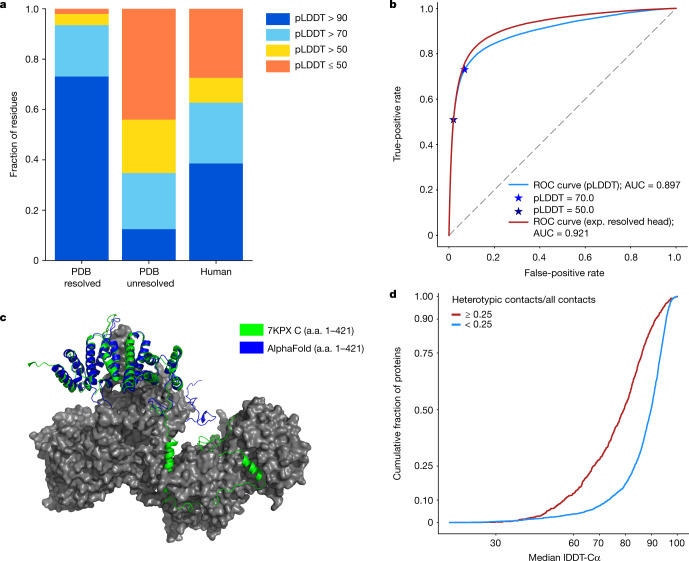
Low-confidence regions. **a**, pLDDT distribution of the resolved parts of PDB sequences (*n* = 3,440,359 residues), the unresolved parts of PDB sequences (*n* = 589,079 residues) and the human proteome (*n* = 10,537,122 residues). **b**, Performance of pLDDT and the experimentally resolved head of AlphaFold as disorder predictors on the CAID Disprot-PDB benchmark dataset (*n* = 178,124 residues). **c**, An example low-confidence prediction aligned to the corresponding PDB submission (7KPX chain C)^66^. The globular domain is well-predicted but the extended interface exhibits low pLDDT and is incorrect apart from some of the secondary structure. a.a., amino acid. **d**, A high ratio of heterotypic contacts is associated with a lower AlphaFold accuracy on the recent PDB dataset, restricted to proteins with fewer than 40% of residues with template identity above 30% (*n* = 3,007 chains) (Methods). The ratio of heterotypic contacts is defined as: heterotypic/(intra-chain + homomeric + heterotypic).

Furthermore, we observed a large difference in the pLDDT distribution between resolved and unresolved residues in PDB sequences (Fig. 4a). To investigate this connection, we evaluated pLDDT as a disorder predictor on the Critical Assessment of protein Intrinsic Disorder prediction (CAID) benchmark dataset^25^. The results showed pLDDT to be a competitive disorder predictor compared with the current state of the art (SPOT-Disorder2^63^), with an area under the curve (AUC) of 0.897 (Fig. 4b). Moreover, the supplementary information of the companion AlphaFold paper^2^ describes an ‘experimentally resolved head’, which is specifically trained for the task of predicting whether a residue will be resolved in an experimental structure. The experimentally resolved head performed even better on the CAID benchmark, with an AUC of 0.921.

These disorder prediction results suggest that a considerable percentage of low-confidence residues may be explained by some form of disorder, but we caution that this could encompass both regions that are intrinsically disordered and regions that are structured only in complex. A potential example of the latter scenario drawn from a recent PDB structure is shown in Fig. 4c; chain C interacts extensively with the rest of the complex, such that the interface region would be unlikely to adopt the same structure outside of this context. In a systematic analysis of recent PDB chains, we observed that AlphaFold has much lower accuracy for regions in which the chain has a high percentage of heterotypic, cross-chain contacts (Fig. 4d).

In summary, our current interpretation of regions in which AlphaFold exhibits low pLDDT is that they have high likelihood of being unstructured in isolation. In the current dataset, long regions with pLDDT < 50 adopt a readily identifiable ribbon-like appearance, and should not be interpreted as structures but rather as a prediction of disorder.

## Discussion

In this study, we generated comprehensive, state-of-the-art structure predictions for the human proteome. The resulting dataset makes a large contribution to the structural coverage of the proteome; particularly for tasks in which high accuracy is advantageous, such as molecular replacement or the characterization of binding sites. We also applied several metrics produced by building on the AlphaFold architecture—pLDDT, pTM and the experimentally resolved head—to demonstrate how they can be used to interpret our predictions.

Although we present several case studies to illustrate the type of insights that may be gained from these data, we recognize that there is still much more to uncover. By making our predictions available to the community via https://alphafold.ebi.ac.uk/, we hope to enable exploration of new directions in structural bioinformatics.

The parts of the human proteome that are still without a confident prediction represent directions for future research. Some proportion of these will be genuine failures, in which a fixed structure exists but the current version of AlphaFold does not predict it. In many other cases, in which the sequence is unstructured in isolation, the problem arguably falls outside the scope of single-chain structure prediction. It will be crucial to develop new methods that can address the biology of these regions—for example, by predicting the structure in complex or by predicting a distribution over possible states in the cellular milieu.

Finally, we note that the importance of the human proteome for health and medicine has led to it being intensively studied from a structural perspective. Other organisms are much less well represented in the PDB, including biologically important, medically relevant or economically important species. Structure prediction may have a more profound effect on the study of these organisms, for which fewer experimental structures are available. Looking beyond the proteome scale, the UniProt database contains hundreds of millions of proteins that have so far been addressed mainly by sequence-based methods, and for which the easy availability of structures could open up entirely new avenues of investigation. By providing scalable structure prediction with very high accuracy, AlphaFold could enable an exciting shift towards structural bioinformatics, further illuminating protein space.

## Methods

### Structure prediction (human proteome)

Sequences for the human reference proteome were obtained from UniProt release 2021_02^6^. Structure prediction was attempted for all sequences with 16–2,700 amino acids; sequences with residue codes B, J, O, U, Z or X were excluded. The length ceiling of 2,700 residues does not represent an absolute limit for the method, but was chosen to keep run times manageable. The structure prediction process was largely as described in the AlphaFold paper^2^, consisting of five steps: MSA construction, template search, inference with five models, model ranking based on mean pLDDT and constrained relaxation of the predicted structures. The following differences were introduced for the proteome-scale pipeline. First, the search against the metagenomics database Big Fantastic Database (BFD) was replaced with a search against ‘Reduced BFD’ using Jackhmmer from HMMER3^67,68^. Reduced BFD consists of a multiline FASTA file containing the first non-consensus sequence from each BFD a3m alignment. Second, the amount of ensembling was reduced by a factor of eight. At least four relaxed full chain models were successfully produced for 20,296 sequences out of 20,614 FASTA entries, covering 98.5% of proteins. Sequences with more than 2,700 residues account for the majority of exclusions. This amounts to 10,537,122 residues (92.5% of residues).

### Structure prediction (recent PDB dataset)

For structure predictions of recent PDB sequences, we used a copy of the PDB downloaded on 15 February 2021. Structures were filtered to those with a release date after 30 April 2018 (the date limit for inclusion in the training set). Chains were then further filtered to remove sequences that consisted of a single amino acid, sequences with an ambiguous chemical component at any residue position and sequences without a PDB 40% sequence clustering. Exact duplicates were removed by choosing the chain with the most resolved Cα atoms as the representative sequence. Then, structures with fewer than 16 resolved residues, with unknown residues and structures solved by NMR methods were filtered out. Structure prediction then followed the same procedure as for the human proteome with the same length and residue limits, except that templates with a release date after 30 April 2018 were disallowed. Finally, the dataset was redundancy reduced, by taking the chain with the best non-zero resolution from each cluster in the PDB 40% sequence clustering, producing a dataset of 12,494 chains. This is referred to as the recent PDB dataset.

### Computational resources

Inference was run on V100 graphics processing units (GPUs), with each sequence inferenced five times to produce five inputs to model selection. To prevent out-of-memory errors, long sequences were assigned to multi-GPU workers. Specifically, sequences of length 1,401–2,000 residues were processed by workers with two GPUs, and those of length 2,001–2,700 residues by workers with four GPUs (further details of unified memory on longer proteins are provided in the companion paper^2^; it is possible higher memory workers could be used without additional GPUs).

**Extended Data Fig. 6:**
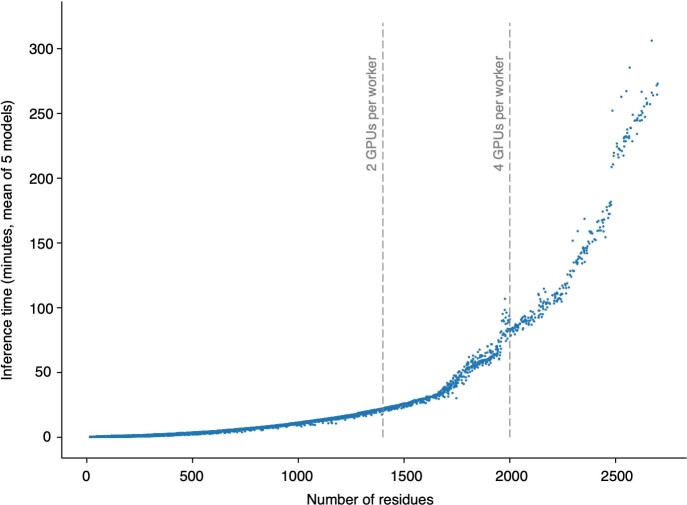
Relationship between sequence length and inference time. On the basis of logs from our human proteome set. All of the processed proteins are shown (*n* = 20,296). Each point indicates the mean inference time for the protein over the models produced. Vertical lines show the length cut-offs above which sequences were processed by multi-GPU workers.

The total resources used for inference were logged and amounted to 930 GPU days. This accounts for generating five models per protein; around 190 GPU days would be sufficient to inference each protein once. Long sequences had a disproportionate effect owing to the multi-GPU workers described above. Approximately 250 GPU days would have been sufficient to produce five models for all proteins shorter than 1,400 residues. For reference, Extended Data Fig. 6 shows the relationship between sequence length and inference time.

**Extended Data Fig. 7:**
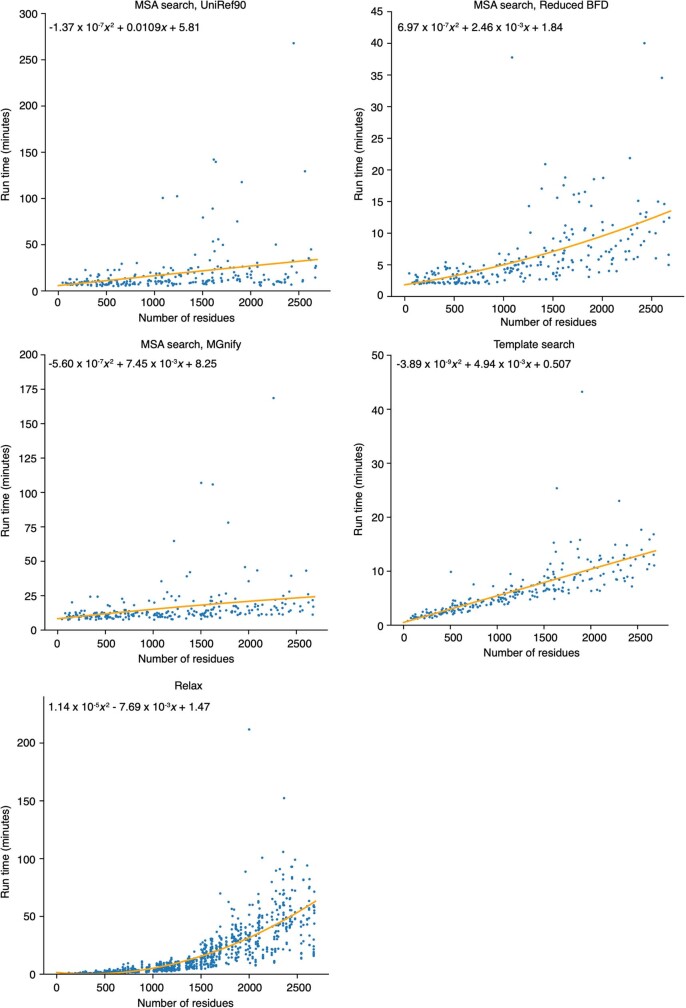
Relationship between sequence length and run time for the non-inference stages of the pipeline. On the basis of 240 human protein sequences, chosen by stratified sampling from the length buckets: [16, 500), [500, 1,000), [1,000, 1,500), [1,500, 2,000), [2,000, 2,500) and [2,500, 2,700]. The relax plot shows five times more points, since five relaxed models are generated per protein. Coefficients for the quadratic lines of best fit were computed with Numpy polyfit.

All other stages of the pipeline (MSA search, template search and constrained relaxation) ran on the central processing unit (CPU) and used standard tools. Our human proteome run made use of some cached intermediates (for example, stored MSA search results). However, we estimate the total cost of running these stages from scratch at 510 core days. This estimate is based on taking a sample of 240 human proteins stratified by length, timing each stage when run with empty caches, fitting a quadratic relationship between sequence length and run time, then applying that relationship to the sequences in the human proteome. Extended Data Figure 7 shows the data used to make this estimate.

### Template coverage

Except where otherwise noted, template coverage was estimated on a per-residue basis as follows. Hmmsearch was run against a copy of the PDB SEQRES (downloaded on 15 February 2021) using default flags^67^. The prior template coverage at residue *i* is the maximum percentage sequence identity of all hits covering residue *i*, regardless of whether the hit residue is experimentally resolved. For the recent PDB analysis, only template hits corresponding to a structure released before 30 April 2018 were accepted.

In the section on full chain prediction, template filtering is based on the highest sequence identity of any single Hmmsearch hit with more than 50% coverage. This is because high-coverage templates are particularly relevant when considering whether a predicted domain packing is novel.

### GO term breakdown

GO annotations were taken from the XML metadata for the UniProt human reference proteome and were matched to the Gene Ontology in obo format^36,37^. One erroneous is_a relationship was manually removed (GO:0071702 is_a GO:0006820, see change log https://www.ebi.ac.uk/QuickGO/term/GO:0071702). The ontology file was used to propagate the GO annotations using is_a and part_of relations to assign parent–child relationships, and accounting for alternative IDs.

GO terms were then filtered to a manageable number for display, first by filtering for terms with more than 3,000 annotations, and from those selecting only moderately specific terms (a term cannot have a child with more than 3,000 annotations). The remaining terms in the ‘molecular function’ and ‘cellular component’ ontologies are shown in Fig. 1d.

### Structure analysis

Structure images were created in PyMOL^69^, and PyMOL align was used to compute r.m.s.d.s (outlier rejection is described in the text where applicable).

For docking against DGAT2, P2Rank^65^ was used to identify ligand-binding pockets in the AlphaFold structure. AutoDockTools^70^ was used to convert the AlphaFold prediction to PDBQT format. For the ligands, DGAT2-specific inhibitor (CAS number 1469284-79-4) and DGAT1-specific inhibitor (CAS number 942999-61-3) were also prepared in PDBQT format using AutoDockTools. AutoDock Vina^71^ was run with an exhaustiveness parameter of 32, a seed of 0 and a docking search space of 25 × 25 × 25 Å^3^ centred at the point identified by P2Rank.

For identifying the most similar structure to wolframin, TM-align^42^ was used to compare against all PDB chains (downloaded 15 February 2021) with our prediction as the reference. This returned 3F1Z with a TM-score of 0.472.

### Additional metrics

The implementation of pTM is described in supplementary information section 1.9.7 of the companion AlphaFold paper^2^ and the implementation of the experimentally resolved head is described in supplementary information section 1.9.10 of the companion AlphaFold paper^2^. The weighted version of pTM is described in Supplementary Methods 1.

### Analysis of low-confidence regions

For evaluation on CAID, the target sequences and ground-truth labels for the Disprot-PDB dataset were downloaded from https://idpcentral.org/. Structure prediction was performed as described above for the recent PDB dataset, with a template cut-off of 30 April 2018. To enable complete coverage, two sequences containing non-standard residues (X, U) had these remapped to G (glycine). Sequences longer than 2,000 residues were split into two segments: 1–2,000 and 2,000–end, and the pLDDT and experimentally resolved head arrays were concatenated for evaluation. The two evaluated disorder predictors were taken to be 1 −0.01 × pLDDT and 1 − predicted resolvability for Cα atoms.

To obtain the ratio of heterotypic contacts to all contacts (Fig. 4d), two residues are considered in contact if their Cβ atoms (or Cα for glycine) are within 8 Å and if they are separated in primary sequence by at least three other residues (to exclude contacts within an α-helix). Heteromers are identified as protein entities with a different entity_id in the structure mmCIF file.

### Comparison with BestSingleStructuralTemplate

CAMEO data for the period 21 March 2020 to 13 March 2021 were downloaded from the CAMEO website. AlphaFold predictions were produced for all sequences in the target.fasta files, using the same procedure detailed above but with a maximum template date of 1 March 2020. Predictions were scored against the CAMEO ground truth using lDDT-Cα. For BestSingleStructuralTemplate, lDDT-Cα scores were taken from the CAMEO JavaScript Object Notation (JSON) files provided. Structures solved by solution NMR and solid-state NMR were filtered out at the analysis stage. To determine the template identity, templates were drawn from a copy of the PDB downloaded on 15 February 2021 with a template search performed using Hmmsearch. Templates were filtered to those with at least 70% coverage of the sequence and a release date before the query. The template with the highest *e*-value after filtering was used to compute the template identity. Targets were binned according to template identity, with width 10 bins ranging from 30 to 90. Extended Data Figure 4 shows the distribution of lDDT-Cα for each model within each bin as a box plot (horizontal line at the median, box spanning from the lower to the upper quartile, whiskers extending to the minimum and maximum. In total 428 targets were included in the analysis.

### Reporting summary

Further information on research design is available in the Nature Research Reporting Summary linked to this paper.

## Online content

Any methods, additional references, Nature Research reporting summaries, source data, extended data, supplementary information, acknowledgements, peer review information; details of author contributions and competing interests; and statements of data and code availability are available at 10.1038/s41586-021-03828-1.

### Supplementary information

Supplementary Methods This file contains (1) Predicted TM-score weighting, and (2) DGAT docking scores.

Reporting Summary

Supplementary Video 1 Solvent accessibility of putative active site for G6Pase-α.

### Source data

Source Data Extended Data Fig. 4

## Figures and Tables

**Extended Data Table 1:**
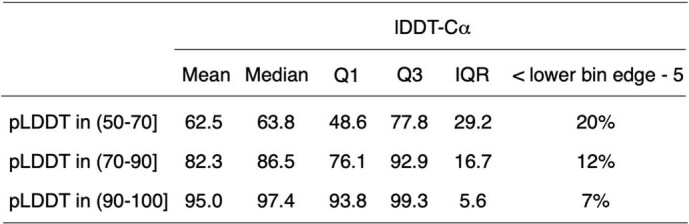
lDDT-Cα distribution in various pLDDT bins

Data are based on the per-residue lDDT-Cα and per-residue pLDDT of resolved regions. This table uses the recent PDB dataset (Methods), which is restricted to structures with a reported resolution of <3.5 Å. The total number of chains included is 10,215.

**Extended Data Table 2:**
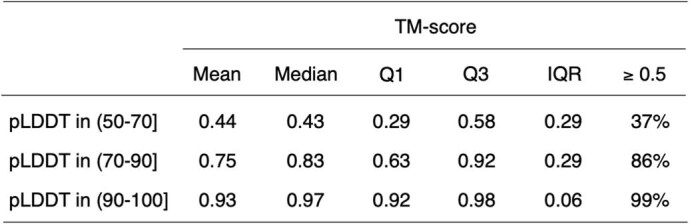
Relationship between pLDDT and TM-score

Binning is based on the mean pLDDT over each chain, weighted by the output of the experimentally resolved head. This table uses the recent PDB dataset (Methods), which restricted to structures with a reported resolution of <3.5 Å. The total number of chains included is 10,215.

## Data Availability

Structure predictions by AlphaFold for the human proteome are available under a CC-BY-4.0 license at https://alphafold.ebi.ac.uk/. All input data are freely available from public sources. The human reference proteome together with its XML annotations was obtained from UniProt v.2021_02 (https://ftp.ebi.ac.uk/pub/databases/uniprot/previous_releases/release-2021_02/knowledgebase/). At prediction time, MSA search was performed against UniRef90 v.2020_03 (https://ftp.ebi.ac.uk/pub/databases/uniprot/previous_releases/release-2020_03/uniref/), MGnify clusters v.2018_12 (https://ftp.ebi.ac.uk/pub/databases/metagenomics/peptide_database/2018_12/) and a reduced version of BFD (produced as outlined in the Methods using the BFD (https://bfd.mmseqs.com/)). Template structures, the SEQRES fasta file and the 40% sequence clustering were taken from a copy of the PDB downloaded on 15 February 2021 (https://www.wwpdb.org/ftp/pdb-ftp-sites; see also https://ftp.wwpdb.org/pub/pdb/derived_data/ and https://cdn.rcsb.org/resources/sequence/clusters/bc-40.out for sequence data). Experimental structures were drawn from the same copy of the PDB; we show structures with accessions 6YJ1^64^, 6OFS^43^, 1IDQ^46^, 1PRT^72^, 3F1Z^58^, 7KPX^66^ and 6VP0^51^. The template search used PDB70, downloaded on 10 February 2021 (http://wwwuser.gwdg.de/~compbiol/data/hhsuite/databases/hhsuite_dbs/). The CAID dataset was downloaded from https://idpcentral.org/caid/data/1/reference/disprot-disorder-pdb-atleast.txt. CAMEO data was accessed on 17 March 2021 at https://www.cameo3d.org/static/downloads/modeling/1-year/raw_targets-1-year.public.tar.gz. A copy of the current Gene Ontology database was downloaded on 29 April 2021 from http://current.geneontology.org/ontology/go.obo. Source data are provided with this paper.

## Code availability

Source code for the AlphaFold model, trained weights and an inference script are available under an open-source license at https://github.com/deepmind/alphafold. Neural networks were developed with TensorFlow v.1 (https://github.com/tensorflow/tensorflow), Sonnet v.1 (https://github.com/deepmind/sonnet), JAX v.0.1.69 (https://github.com/google/jax/) and Haiku v.0.0.4 (https://github.com/deepmind/dm-haiku).

 For MSA search on UniRef90, MGnify clusters and the reduced BFD, we used jackhmmer and for the template search on the PDB SEQRES we used hmmsearch, both from HMMER v.3.3 (http://eddylab.org/software/hmmer/). For the template search against PDB70, we used HHsearch from HH-suite v.3.0-beta.3 14/07/2017 (https://github.com/soedinglab/hh-suite). For constrained relaxation of structures, we used OpenMM v.7.3.1 (https://github.com/openmm/openmm) with the Amber99sb force field.

 Docking analysis on DGAT used P2Rank v.2.1 (https://github.com/rdk/p2rank), MGLTools v.1.5.6 (https://ccsb.scripps.edu/mgltools/) and AutoDockVina v.1.1.2 (http://vina.scripps.edu/download/) on a workstation running Debian GNU/Linux rodete 5.10.40-1rodete1-amd64 x86_64.

 Data analysis used Python v.3.6 (https://www.python.org/), NumPy v.1.16.4 (https://github.com/numpy/numpy), SciPy v.1.2.1 (https://www.scipy.org/), seaborn v.0.11.1 (https://github.com/mwaskom/seaborn), scikit-learn v.0.24.0 (https://github.com/scikit-learn/), Matplotlib v.3.3.4 (https://github.com/matplotlib/matplotlib), pandas v.1.1.5 (https://github.com/pandas-dev/pandas) and Colab (https://research.google.com/colaboratory). TM-align v.20190822 (https://zhanglab.dcmb.med.umich.edu/TM-align) was used for computing TM-scores. Structure analysis used Pymol v.2.3.0 (https://github.com/schrodinger/pymol-open-source).
