# Supplementary material for: Highly accurate protein structure prediction for the human proteome
Source: Nature. 2021 Jul 22;596(7873):590–6. doi: 10.1038/s41586-021-03828-1 (PMC8387240; doi:10.1038/s41586-021-03828-1)
Supplement: Supplementary file 1 — This file contains (1) Predicted TM-score weighting, and (2) DGAT docking scores. [file 41586_2021_3828_MOESM1_ESM.pdf]

nature portfolio

https://doi.org/10.1038/s41586-021-03828-1

Supplementary information

Highly accurate protein structure prediction for the human proteome

In the format provided by the authors and unedited

Supplementary Information for Highly accurate protein structure prediction for the human proteome

Kathryn Tunyasuvunakool[1 †], Jonas Adler[1], Zachary Wu[1], Tim Green[1], Michal Zielinski[1], Augustin Žídek[1], Alex Bridgland[1], Andrew Cowie[1], Clemens Meyer[1], Agata Laydon[1], Sameer Velankar[2], Gerard J Kleywegt[2], Alex Bateman[2], Richard Evans[1], Alexander Pritzel[1], Michael Figurnov[1], Olaf Ronneberger[1], Russ Bates[1], Simon A A Kohl[1], Anna Potapenko[1], Andrew J Ballard[1], Bernardino Romera-Paredes[1], Stanislav Nikolov[1], Rishub Jain[1], Ellen Clancy[1], David Reiman[1], Stig Petersen[1], Andrew W Senior[1], Koray Kavukcuoglu[1], Ewan Birney[2], Pushmeet Kohli[1], John Jumper[1* †], Demis Hassabis[1* †]

[1]DeepMind, London, UK
[2]European Molecular Biology Laboratory, European Bioinformatics Institute, Hinxton, UK
[*]Equal contribution: John Jumper, Demis Hassabis
[†] e-mail: ktkool@deepmind.com, jumper@deepmind.com, dhcontact@deepmind.com

Table of Contents

Supplementary Methods

Section 1: Predicted TM-score weighting

Section 2: DGAT docking scores

Supplementary Files

Supplementary Video 1: Solvent accessibility of putative active site for G6Pase-α

Source Data: PDB ID and author chain ID for the 428 CAMEO targets used to generate Extended Data Fig. 4

Supplementary Methods

Section 1: Predicted TM-score weighting

The AlphaFold paper SI (Section 1.9.7) describes pTM, an estimate of the TM-score:

$$\mathrm{pTM} = \max_{i \in [1,\ldots,N_{\mathrm{res}}]} \frac{1}{N_{\mathrm{res}}} \sum_{j=1}^{N_{\mathrm{res}}} \mathbb{E}\left[f(e_{ij})\right],$$

where N_{res} is the number of residues in the prediction, $f(d) = 1 / (1 + (d / d_0(N_{res}))^2)$ is the TM-score kernel function that transforms distance errors to the range [0, 1], and e_{ij} is the estimate produced by the neural network of the distance error for residue *j*'s Cα in the backbone frame of residue *i*. The expectation is over the probability distribution of e_{ij}.

We find that this expression can produce pessimistic results when some part of the prediction is unstructured. In particular, it may not reflect the model's confidence about the packing of the structured domains that *are* present. To alleviate this, we develop a weighted version that modulates the contribution of each residue by the estimated probability that this residue's Cα atom will be experimentally resolved (see “experimentally resolved head”, AlphaFold paper SI Section 1.9.10). Denoting this probability by p_j, the weighted pTM is:

$$\begin{aligned} a_i &= \frac{1}{\sum_k p_k} \sum_{j=1}^{N_{\mathrm{res}}} p_j \cdot \mathbb{E}\left[f(e_{ij})\right] \\ i^* &= \mathrm{argmax}_i \, a_i \cdot p_i \\ \mathrm{pTM}_{\mathrm{weighted}} &= a_{i^*} \end{aligned}$$

We also replace N_{res} with $\sum_j p_j$, the expected number of resolved residues, in the definition of $f(d)$. In the first line, we compute the value of TM-score when aligning on each residue, while taking into account the probability of all other residues being experimentally resolved. In the second line, we choose the residue i^* that is expected to produce the optimal alignment, while

penalizing alignments on potentially unresolved residues. In the final line, we report the value of the TM-score for this chosen alignment.

If the probability of being experimentally resolved p_j only takes the values 0 or 1, then $pTM_{weighted}$ simply becomes the pTM evaluated on a subset of the prediction corresponding to $p_j=1$.

The 2D images of the expected values $\mathbb{E}[e_{ij}]$, which we call the Predicted Aligned Error, are useful visualizations of the model's domain packing confidence. The value in position (i, j) is the expected error in residue j's position, when both the prediction and the ground truth are aligned on residue i. Confident domain packings will have low errors in the off-diagonal regions corresponding to the interaction of the two domains. Note that these maps are asymmetric and sensitive to the orientation of residue i, but not j. For example, if residue i is part of a mobile loop of the prediction, then we can expect high errors in the whole row (i, j) corresponding to this residue. However, the converse is not always true: the error (j, i) may be quite high even when the error in position (i, j) is low.

Section 2: DGAT docking scores

To explore whether our docking scores correlate with biological assays, we further considered docking inhibitors specific to DGAT1 and DGAT2 to both receptors. The DGAT1[49] and DGAT2[48] inhibitors show over 1000-fold differences in IC_{50} under published conditions, and we hypothesized that this large difference would show some signal in the docking score.

To this end, we performed rigid receptor docking of the two inhibitors against predicted DGAT2, predicted DGAT1, and ground truth DGAT1 (PDB: 6VP0). We also tested flexible receptor docking of the two inhibitors, but observed high variability in docking scores with different seeds, even after increasing the exhaustiveness to 64, and proceeded with rigid receptors.

Docking the inhibitors to the predicted DGAT2 structure, we observed a 1.6 kcal/mol stronger predicted docking score for the DGAT2 inhibitor. On the ground truth DGAT1 structure, docking predicts a 0.5 kcal/mol stronger docking score for the DGAT1 inhibitor. However, on the predicted DGAT1 structure, we observed a 0.2 kcal/mol stronger docking score for the DGAT2 inhibitor. While the relative docking scores of the two inhibitors on the predicted DGAT1 structure do not match our expectation, these differences in docking scores are well within the 2.8 kcal/mol standard error published for Autodock Vina[71]. As Vina was developed for accuracy in binding pose (and not docking score), we caution against interpreting docking scores as binding affinities, as do others[76].

On examination of the top docking poses of the DGAT2 inhibitor on DGAT2, the core imidazopyridine ring sits comfortably between the proposed catalytic His163 and polar Thr194, where it is well-placed for hydrogen bond interactions (**Fig. 3b**). Thr194 may also form hydrogen bonds with the inhibitor's pyrazole group, which is constrained by the joining cyclopropyl motif. In contrast, the best binding pose for the docked DGAT1 inhibitor binds to an alternate region on the outside of the protein (**Extended Data Fig. 5a**), which P2Rank predicts to have a low ligandability score. The next best pose for the DGAT1 inhibitor matches the predicted DGAT2 inhibitor binding site (**Extended Data Fig. 5b**), but the DGAT1 inhibitor has no polar groups in its core to satisfy His163 and Thr194 in the best binding pose. A comparison of the two pockets

is shown in **Extended Data Fig. 5c**. Thus, the docked predicted binding poses are consistent with experimental evidence.

References

76. Fischer, A., Smiesko, M., Sellner, M. & Lill, M. A. Decision making in structure-based drug discovery: visual inspection of docking results. *J. Med. Chem.* **64**, 2489-2500 (2021).
