# Supplementary material for: Highly accurate protein structure prediction for the human proteome
Source: Nature. 2021 Jul 22;596(7873):590–6. doi: 10.1038/s41586-021-03828-1 (PMC8387240; doi:10.1038/s41586-021-03828-1)
Supplement: Supplementary file 2 — Reporting Summary [file 41586_2021_3828_MOESM2_ESM.pdf]

nature research

Corresponding author(s): Kathryn Tunyasuvunakool, John Jumper, Demis Hassabis

Last updated by author(s): Jul 13, 2021

Reporting Summary

Nature Research wishes to improve the reproducibility of the work that we publish. This form provides structure for consistency and transparency in reporting. For further information on Nature Research policies, see our Editorial Policies and the Editorial Policy Checklist.

Statistics

For all statistical analyses, confirm that the following items are present in the figure legend, table legend, main text, or Methods section.

n/a	Confirmed	
☐	☒	The exact sample size (*n*) for each experimental group/condition, given as a discrete number and unit of measurement
☒	☐	A statement on whether measurements were taken from distinct samples or whether the same sample was measured repeatedly
☒	☐	The statistical test(s) used AND whether they are one- or two-sided<br>*Only common tests should be described solely by name; describe more complex techniques in the Methods section.*
☒	☐	A description of all covariates tested
☒	☐	A description of any assumptions or corrections, such as tests of normality and adjustment for multiple comparisons
☐	☒	A full description of the statistical parameters including central tendency (e.g. means) or other basic estimates (e.g. regression coefficient) AND variation (e.g. standard deviation) or associated estimates of uncertainty (e.g. confidence intervals)
☒	☐	For null hypothesis testing, the test statistic (e.g. *F*, *t*, *r*) with confidence intervals, effect sizes, degrees of freedom and *P* value noted<br>*Give P values as exact values whenever suitable.*
☒	☐	For Bayesian analysis, information on the choice of priors and Markov chain Monte Carlo settings
☒	☐	For hierarchical and complex designs, identification of the appropriate level for tests and full reporting of outcomes
☐	☒	Estimates of effect sizes (e.g. Cohen's *d*, Pearson's *r*), indicating how they were calculated

Our web collection on statistics for biologists contains articles on many of the points above.

Software and code

Policy information about availability of computer code

Data collection	Source code for the AlphaFold model, trained weights, and an inference script are available under an open-source license at https://github.com/deepmind/alphafold. Neural networks were developed with TensorFlow v1 (https://github.com/tensorflow/tensorflow), Sonnet v1 (https://github.com/deepmind/sonnet), JaX v0.1.69 (https://github.com/google/jax/), and Haiku v0.0.4 (https://github.com/deepmind/dm-haiku).<br><br>For MSA search on UniRef90, MGnify clusters, and reduced BFD we used jackhmmer and for template search on the PDB seqres we used hmmsearch, both from HMMER v3.3 (http://eddylab.org/software/hmmer/). For template search against PDB70, we used HHsearch from HH-suite v3.0-beta.3 14/07/2017 (https://github.com/soedinglab/hh-suite). For constrained relaxation of structures, we used OpenMM v7.3.1 (https://github.com/openmm/openmm) with the Amber99sb force field.<br><br>Docking analysis on DGAT used P2Rank v2.1 (https://github.com/rdk/p2rank), MGLTools v1.5.6 (https://ccsb.scripps.edu/mgltools/) and AutoDockVina v1.1.2 (http://vina.scripps.edu/download/) on a workstation running Debian GNU/Linux rodete 5.10.40-1rodete1-amd64 x86_64.
Data analysis	Data analysis used Python v3.6 (https://www.python.org/), NumPy v1.16.4 (https://github.com/numpy/numpy), SciPy v1.2.1 (https://www.scipy.org/), seaborn v0.11.1 (https://github.com/mwaskom/seaborn), scikit-learn v0.24.0 (https://github.com/scikit-learn/), Matplotlib v3.3.4 (https://github.com/matplotlib/matplotlib), pandas v1.1.5 (https://github.com/pandas-dev/pandas), and Colab (https://research.google.com/colaboratory). TM-align v20190822 (https://zhanglab.dcmb.med.umich.edu/TM-align) was used for computing TM-scores. Structure analysis used Pymol v2.3.0 (https://github.com/schrodinger/pymol-open-source).

For manuscripts utilizing custom algorithms or software that are central to the research but not yet described in published literature, software must be made available to editors and reviewers. We strongly encourage code deposition in a community repository (e.g. GitHub). See the Nature Research guidelines for submitting code & software for further information.

Data

Policy information about availability of data

All manuscripts must include a data availability statement. This statement should provide the following information, where applicable:

- Accession codes, unique identifiers, or web links for publicly available datasets
- A list of figures that have associated raw data
- A description of any restrictions on data availability

AlphaFold structure predictions for the human proteome are available under a CC-BY-4.0 license at https://alphafold.ebi.ac.uk/.

All input data are freely available from public sources. The human reference proteome together with its xml annotations was obtained from UniProt 2021_02 (https://ftp.ebi.ac.uk/pub/databases/uniprot/previous_releases/release-2021_02/knowledgebase/).
At prediction time, MSA search was performed against UniRef90 2020_03 (https://ftp.ebi.ac.uk/pub/databases/uniprot/previous_releases/release-2020_03/uniref/), MGnify clusters 2018_12 (https://ftp.ebi.ac.uk/pub/databases/metagenomics/peptide_database/2018_12/), and a reduced version of BFD (produced from as outlined in the Methods from BFD https://bfd.mmseqs.com/). Template structures, the SEQRES fasta file, and the 40% sequence clustering were taken from a copy of the PDB downloaded 15/2/2021 (https://www.wwpdb.org/ftp/pdb-ftp-sites; see also https://ftp.wwpdb.org/pub/pdb/derived_data/ and https://cdn.rcsb.org/resources/sequence/clusters/bc-40.out for sequence data). Experimental structures are drawn from the same copy of the PDB; we show structures with accessions 6YJ1, 6OFS, 1IDQ, 1PRT, 3F1Z, 7KPX and 6VP0. Template search used PDB70, downloaded 10/02/2021 (http://wwwuser.gwdg.de/~compbiol/data/hhsuite/databases/hhsuite_dbs/). The CAID dataset was downloaded from (https://idpcentral.org/caid/data/1/reference/disprot-disorder-pdb-atleast.txt). CAMEO data was accessed 17/03/2021 from (https://www.cameo3d.org/static/downloads/modeling/1-year/raw_targets-1-year.public.tar.gz). A copy of the current Gene Ontology was downloaded 29/04/2021 from (http://current.geneontology.org/ontology/go.obo).

Field-specific reporting

Please select the one below that is the best fit for your research. If you are not sure, read the appropriate sections before making your selection.

☒ Life sciences ☐ Behavioural & social sciences ☐ Ecological, evolutionary & environmental sciences

For a reference copy of the document with all sections, see nature.com/documents/nr-reporting-summary-flat.pdf

Life sciences study design

All studies must disclose on these points even when the disclosure is negative.

Sample size	This study does not concern a sample; the main results concern the largest subset of the human reference proteome that it was easily practical to inference.
Data exclusions	Proteins were excluded from the analysis for length reasons (less than 16 or greater than 2700 residues), or because they contained a nonstandard single letter amino acid code in their fasta sequence.
Replication	Not applicable, no experimental work is described in this study. The results are the output of a computational method which will be made available.
Randomization	Not applicable, we are not drawing a comparison between two groups
Blinding	Not applicable, we are not drawing a comparison between two groups

Reporting for specific materials, systems and methods

We require information from authors about some types of materials, experimental systems and methods used in many studies. Here, indicate whether each material, system or method listed is relevant to your study. If you are not sure if a list item applies to your research, read the appropriate section before selecting a response.

Materials & experimental systems

n/a	Involved in the study	
☒	☐	Antibodies
☒	☐	Eukaryotic cell lines
☒	☐	Palaeontology and archaeology
☒	☐	Animals and other organisms
☒	☐	Human research participants
☒	☐	Clinical data
☒	☐	Dual use research of concern

Methods

n/a	Involved in the study	
☒	☐	ChIP-seq
☒	☐	Flow cytometry
☒	☐	MRI-based neuroimaging
